# Serum heme oxygenase-1 measurement is useful for evaluating disease activity and outcomes in patients with acute respiratory distress syndrome and acute exacerbation of interstitial lung disease

**DOI:** 10.1186/s12890-020-01341-1

**Published:** 2020-11-25

**Authors:** Ryo Nagasawa, Yu Hara, Kota Murohashi, Ayako Aoki, Nobuaki Kobayashi, Shigeto Takagi, Satoru Hashimoto, Akihiko Kawana, Takeshi Kaneko

**Affiliations:** 1 Department of Pulmonology, Yokohama City University Graduate School of Medicine, 3-9 Fukuura, Kanazawa-ku, Yokohama City, 236-0004 Japan; 2 Seamen’s Insurance Health Management Center, Yokohama, Japan; 3 Division of Intensive Care Unit, Kyoto Prefectural University of Medicine, Kyoto, Japan; 4 Division of Infectious Diseases and Pulmonary Medicine, Department of Internal Medicine, National Defense Medical College, Saitama, Japan

**Keywords:** Acute respiratory distress syndrome, Heme oxygenase-1, Interstitial lung disease, Lung injury, Oxidative stress, Disease activity, Outcome

## Abstract

**Background:**

Oxidative stress plays an important role in acute lung injury, which is associated with the development and progression of acute respiratory failure. Here, we investigated whether the degree of oxidative stress as indicated by serum heme oxygenase-1 (HO-1) is clinically useful for predicting prognosis among the patients with acute respiratory distress syndrome (ARDS) and acute exacerbation of interstitial lung disease (AE-ILD).

**Methods:**

Serum HO-1 levels of newly diagnosed or untreated ARDS and AE-ILD patients were measured at diagnosis. Relationships between serum HO-1 and other clinical parameters and 1 and 3-month mortality were evaluated.

**Results:**

Fifty-five patients including 22 of ARDS and 33 of AE-ILD were assessed. Serum HO-1 level at diagnosis was significantly higher in ARDS patients than AE-ILD patients (87.8 ± 60.0 ng/mL vs. 52.5 ± 36.3 ng/mL, *P* <  0.001). Serum HO-1 correlated with serum total bilirubin (R = 0.454, P <  0.001) and serum LDH (R = 0.500, P <  0.001). In both patients with ARDS and AE-ILDs, serum HO-1 level tended to decrease from diagnosis to 2 weeks after diagnosis, however, did not normalized. Composite parameters including serum HO-1, age, sex, and partial pressure of oxygen in arterial blood/fraction of inspired oxygen (P/F) ratio for prediction of 3-month mortality showed a higher AUC (ARDS: 0.925, AE-ILDs: 0.892) than did AUCs of a single predictor or combination of two or three predictors.

**Conclusion:**

Oxidative stress assessed by serum HO-1 is persistently high among enrolled patients for 2 weeks after diagnosis. Also, serum HO-1 levels at the diagnosis combined with age, sex, and P/F ratio could be clinically useful for predicting 3-month mortality in both ARDS and AE-ILD patients.

## Introduction

Acute respiratory distress syndrome (ARDS) is one of the major manifestations of multiple organ failure syndrome and is a leading cause of death in intensive care units [1]. Within the clinical course of interstitial lung disease (ILD), an acute exacerbation (AE) can occur at any time and is associated with significant morbidity and mortality [2]. Diffuse alveolar damage (DAD) is considered the histological hallmark of the acute phase of ARDS and AE-ILDs, while alternative histological appearances comprise organizing pneumonia, alveolar haemorrhage, and unspecific inflammatory changes [3, 4]. The clinical course and rate of progression of ARDS and AE-ILDs are extremely variable among patients. Therefore, biomarkers including symptoms, blood, physiological, radiological, and pathological findings and these combinations may be useful in characterizing disease severity and predicting the rate of progression and response to therapies [5, 6].

Oxidative/nitrosative stress results from an imbalance between cellular production of reactive oxygen species (ROS)/reactive nitrogen species (RNS) and the endogenous antioxidants such as stress response protein (heme oxygenase-1 (HO-1)), classic antioxidant enzymes (superoxide dismutases (SODs), catalase, glutathione peroxidase (GPx)) [7]. Nuclear factor-erythroid 2 p45 subunit-related factor 2 (Nrf2) is essential for activating response in the lung due to induction of the expression of antioxidant and these enzymes are expressed in bronchial and alveolar epithelial cells and macrophages of the lung [8]. Several clinical evidences suggested that increased oxidative/nitrosative stress might play a major role in the progression of various lung diseases such as idiopathic pulmonary fibrosis (IPF), chronic obstructive pulmonary disease, and ARDS [9–14]. HO-1 catalyzes heme degradation to biliverdin-IXα, carbon monoxide, and iron. These metabolites mediate the antiapoptotic, anti-inflammatory, vasodilatory, anticoagulant, antioxidant, and antiproliferative properties of HO-1 under the control of the microsomal nicotinamide adenine dinucleotide phosphate-cytochrome p450 reductase [15, 16]. HO-1 expression is induced by various stimuli such as exposure of cigarette smoke extract (CSE), heme, hypoxia, endotoxin, and pro-inflammatory cytokines. Suzuki et al. reported that in human alveolar macrophages, acute CSE exposure increases HO-1 mRNA for 2 h [17]. Mumby et al. reported that HO-1 protein concentrations are significantly elevated in lung tissue and bronchoalveolar lavage fluid taken from ARDS patients compared with controls, and HO-1 expression contributes to changes in iron mobilization, signalling, and regulation seen in this condition [18]. Also, we have demonstrated the usefulness of measuring serum HO-1 in the diagnosis and prognosis of patients with ARDS and AE-ILDs [19, 20]. Therefore, we speculate that HO-1 characterized as rapid stress response protein with various physiological activities caused by HO-1 metabolites could reflect pulmonary cellular damage induced by ROS and RNS more closely and directly [7, 15–17].

In the present study, we investigated whether the degree of oxidative stress measured by serum HO-1 levels at the diagnosis could be useful for predicting prognosis and these levels could decrease during the clinical courses among patients with ARDS and AE-ILDs.

## Methods

### Study location and diagnosis of ARDS asnd AE-ILDs

This multi-institutional prospective study was performed in Yokohama City University, Kyoto Prefectural University, and National Defense Medical College Hospital between 2011 and 2019. We recruited untreated ARDS patients who met the Berlin definition [21]. The diagnosis of idiopathic interstitial pneumonias (IIPs) was confirmed by physical findings, serological testing, high resolution CT (HRCT) finding, and lung biopsy specimens, based on the official statement for IIPs including IPF [22, 23]. Patients whose lung biopsy could not be performed due to severe respiratory failure were diagnosed based on the radiological classification [22, 23]. The diagnosis of collagen vascular disease-related interstitial pneumonia (CVD-IP) was confirmed by physical findings, serological testing, and HRCT findings that were consistent with ILD. AE-ILD patients were defined as having unexplained worsening of dyspnoea; hypoxaemia or worsening or severely impaired gas exchange; new alveolar infiltrates superimposed upon chronic ILD lesions on radiograph; and absence of an alternative explanation such as infection, pulmonary embolism, pneumothorax, or heart failure [24, 25]. In addition, we recruited healthy volunteers among medical personnel of Seamen’s Insurance Health Management Center for health examination.

### Data collection and blood sampling

Extracted data included age, sex, diagnosis including the causes of ARDS, and 1 and 3-month mortality. Blood samples were obtained at the diagnosis of ARDS or AE-ILD from each patient. We measured serum HO-1 along with serum total bilirubin (T-bil; normal range: 0.2–1.2 mg/dL), serum lactate dehydrogenase (LDH; normal range: < 225 U/L), serum C-reactive protein (CRP; normal range: ≤ 0.3 mg/dL), and partial pressure of oxygen in arterial blood/fraction of inspired oxygen (P/F) ratio.

### Serum HO-1 enzyme-linked immunosorbent assay (ELISA) measurement

Serum HO-1 levels were measured at the time of ARDS or AE-ILD diagnosis (D0) and 7 (D7) and 14 (D14) days from the diagnosis using the IMMUNOSET® HO-1 (human) ELISA development set (Enzo, Farmingdale, NY, USA), according to the manufacturer’s instructions. The details of this ELISA method have been described previously [19]. The assay validation was performed reproducibility of ELISA standard curve for serum HO-1, the intra- and inter-assay tests, and the percentage recovery test. We confirmed all of these results were acceptable [19]. Control subjects for serum HO-1 levels included 28 healthy, non-smoking adults who had been admitted to the hospital for a medical checkup.

### Statistical analysis

Data are expressed as mean ± standard deviation. Statistical analysis was performed using JMP11 (SAS Institute, Inc., North Carolina, USA). Group comparisons were made using Wilcoxon’s rank-sum test or the chi-squared test, as appropriate. Spearman’s correlation coefficients were calculated to assess the relationship between serum HO-1 and other clinical parameters. The applicability of serum HO-1 with or without other clinical parameters in predicting 3-month mortality was evaluated using the area under a receiver operating characteristic (ROC) curve (AUC). Survival curves were generated using the Kaplan–Meier method and were compared using the Wilcoxon test. *P* values < 0.05 were considered significant.

## Results

### Patients’ characteristics

**Fig. 1:**
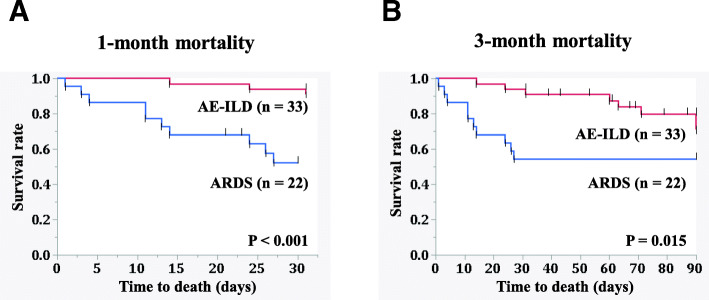
Comparison of 1- and 3-month mortality between ARDS and AE-ILD patients. Among the 55 enrolled patients, 22 were diagnosed with ARDS, and 33 were diagnosed with AE-ILDs. A significant difference in the 1-month mortality rate was evident between ARDS and AE-ILD patients (45% vs. 9%, respectively, *P* < 0.001). Also, a significant difference in the 3-month mortality rate was observed (45% vs. 24%, respectively, P = 0.015)

Table 1 shows the clinical characteristics of patients with ARDS and AE-ILDs. Among the 55 enrolled patients, 22 were diagnosed with ARDS and 33 were diagnosed with AE-ILDs. The causes of ARDS included infection (*n* = 14, 60%) and surgery (*n* = 5, 23%). The diagnosis of ILDs including IIPs (*n* = 21, 64%) and CVD-IP (*n* = 8, 24%) was previously confirmed before the onset of AE. IIPs consisted of 11 patients with IPF patients and 10 patients with non-specific interstitial pneumonia. Among blood biomarkers, serum HO-1 and CRP levels were higher in ARDS patients than in AE-ILD patients (*P* <  0.05). Serum HO-1 in both ARDS and AE-ILD patients were higher than control subjects (9.5 ± 3.3 ng/mL (*n* = 44)). As shown in Fig. 1a, a significant difference in the 1-month mortality rate was evident between ARDS and AE-ILD patients (45% vs. 9%, respectively, *P* <  0.001). Also, as shown in Fig. 1b, a significant difference in the 3-month mortality rate was observed among these patients (45% vs. 24%, respectively, *P* = 0.015).

**Table 1:** Patients’ characteristics

Characteristics	ARDS patients (***n*** = 22)	AE-ILD patients (***n*** = 33)	Total patients (***n*** = 55)	P values (ARDS vs. AE-ILDs)
**Age, y**	66.5 ± 10.2	75.0 ± 8.0	71.4 ± 9.9	0.003
**Male sex**	16 (73)	21 (72)	37 (73)	0.980
**Aetiology of ARDS**				
**Infection**	14 (60)		14 (60)	
**Surgery**	5 (23)		5 (23)	
**Others**	3 (17)		3 (17)	
**Diagnosis of ILDs**				
**IIPs**^a^		21 (64)	21 (64)	
**CVD-IP**		8 (24)	8 (24)	
**Others**		4 (12)	4 (12)	
**Blood biomarkers**				
**P/F ratio**	186.7 ± 103.7	219.4 ± 73.8	204.4 ± 89.3	0.094
**Serum LDH, U/L**	368.5 ± 223.0	332.7 ± 20.9	347.0 ± 168.9	0.993
**Serum HO-1, ng/mL**	87.8 ± 60.0	52.5 ± 36.3	66.6 ± 49.9	0.011
**Serum total bilirubin, mg/dL**	3.9 ± 7.5	0.9 ± 0.4	2.1 ± 5.0	0.090
**Serum CRP, mg/dL**	16.6 ± 10.8	9.4 ± 6.9	12.3 ± 9.3	0.008
**Treatment**				
**Corticosteroid use**	13 (59)	33 (100)	46 (82)	< 0.001
**Corticosteroid pulse use**	3 (14)	27 (82)	30 (55)	< 0.001
**Frequency of PSL pulse**	1	1 (1–4)	1 (1–4)	0.304
**NEI use**	15 (68)	11 (33)	26 (47)	0.011
**Intubation**	22 (100)	0 (0)	22 (40)	< 0.001
**Outcome**				
**1-month mortality**	10 (45)	3 (9)	13 (24)	< 0.001
**3-month mortality**	10 (45)	8 (24)	18 (33)	0.015

Footnotes

Values are reported as mean ± SD or n (%)

^a^IIPs consisted of 11 patients with idiopathic pulmonary fibrosis patients and 10 patients with non-specific interstitial pneumonia

*Abbreviations*: *AE* acute exacerbation, *ARDS* acute respiratory distress syndrome, *CRP* C-reactive protein, *CVD-IP* collagen vascular disease-related interstitial pneumonia, *HO-1* heme oxygenase-1, *IIPs* idiopathic interstitial pneumonias, *ILDs* interstitial lung diseases, *LDH* lactate dehydrogenase, *NEI* neutrophil elastase inhibitor, *P/F ratio* partial pressure of oxygen in arterial blood/fraction of inspired oxygen, *SD* standard deviation

### Serum HO-1 at the baseline (D0) and other blood biomarkers

Serum HO-1 levels were significantly higher in ARDS patients than in AE-ILD patients at D0 (87.8 ± 60.0 ng/mL vs. 52.5 ± 36.3 ng/mL, respectively, P <  0.001). As shown in Table 2, serum HO-1 significantly correlated with serum T-bil (R = 0.454, P <  0.001) and LDH (R = 0.500, P <  0.001), but not with serum CRP and P/F ratio.

**Table 2:** Relationships between serum HO-1 and other blood parameters

Variables	N	R	95% CI	P
**Serum T-bil**	54	0.454	0.212–0.644	< 0.001
**Serum LDH**	55	0.500	0.271–0.676	< 0.001
**Serum CRP**	55	0.262	−0.004–0.493	0.053
**P/F ratio**	48	−0.159	−0.424–0.131	0.281

*CI* confidence interval, *CRP* C-reactive protein, *HO-1* heme oxygenase-1, *LDH* lactate dehydrogenase, *P/F ratio* partial pressure of oxygen in arterial blood/fraction of the inspiratory oxygen; T-bil, total bilirubin

### Variation in serum HO-1 levels (D0, D7, and D14)

**Fig. 2:**
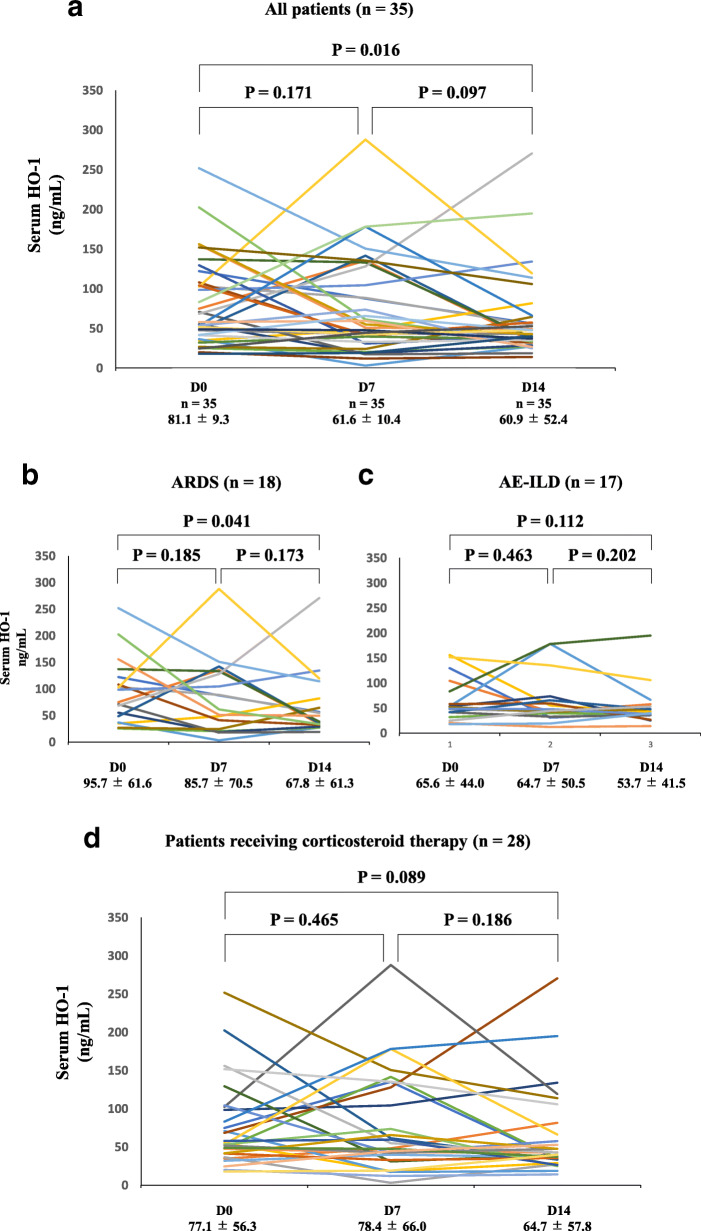
Variation in serum HO-1 levels at the time of diagnosis (D0) and 7 (D7) and 14 (D14) days from the diagnosis. Serum HO-1 levels were measured at the time of ARDS or AE-ILD diagnosis (D0) and 7 (D7) and 14 (D14) days from the diagnosis. Serum HO-1 levels at D0, D7, and D14 were available in 35 of 55 patients (64%). Of the 35 patients, 18 (51%) had ARDS and 17 (49%) had AE-ILDs. As shown in **a** (all patients), serum HO-1 at D0, D7, and D14 tended to decrease, and serum HO-1 levels at D14 were significantly decreased compared with those at D0 (81.1 ± 9.3 ng/mL vs. 60.9 ± 52.4 ng/mL, respectively, P = 0.016). Furthermore, as shown in **b**, significant differences were observed between serum HO-1 levels at D0 and D14 in the ARDS group (95.7 ± 61.6 ng/mL vs. 67.8 ± 61.3 ng/mL, respectively, *P* = 0.041). As shown in **c**, while serum HO-1 levels of the AE-ILD group tended to decrease, these differences were not significant. As shown in **d**, 28 (51%) were treated with corticosteroid and had available HO-1 levels at the time of D0 and D7 and D14 and no significant difference was observed between serum HO-1 levels at D0, D7, and D14 (77.1 ± 56.3 ng/mL vs. 78.4 ± 66.0 ng/mL vs. 64.7 ± 57.8 ng/mL, respectively)

Serum HO-1 levels at D0, D7, and D14 were available in 35 of 55 patients (64%). Of these 35 patients, 18 (51%) patients had ARDS and 17 (49%) patients had AE-ILDs. As shown in Fig. 2a (all patients), serum HO-1 levels tended to decrease over time, and serum HO-1 levels at D14 were significantly lower than those at D0 (81.1 ± 9.3 ng/mL vs. 60.9 ± 52.4 ng/mL, respectively, *P* = 0.016). As shown in Fig. 2b, significant differences were observed between serum HO-1 levels at D0 and D14 in the ARDS patients (95.7 ± 61.6 ng/mL vs. 67.8 ± 61.3 ng/mL, respectively, *P* = 0.041). Although serum HO-1 levels in the AE-ILD patients tended to decrease over time, no significant differences were observed between timepoints (Fig. 2c). Also, we evaluated the variation in serum HO-1 levels in patients treated with corticosteroid. As shown in Fig. 2d, 28 patients (51%) were treated with corticosteroid and had available serum HO-1 levels at D0, D7, and D14. No significant difference was observed between serum HO-1 levels at D0, D7, and D14 (77.1 ± 56.3 ng/mL vs. 78.4 ± 66.0 ng/mL vs. 64.7 ± 57.8 ng/mL, respectively).

### Composite parameters for predicting 3-month mortality in patients with ARDS and AE-ILDs

**Fig. 3:**
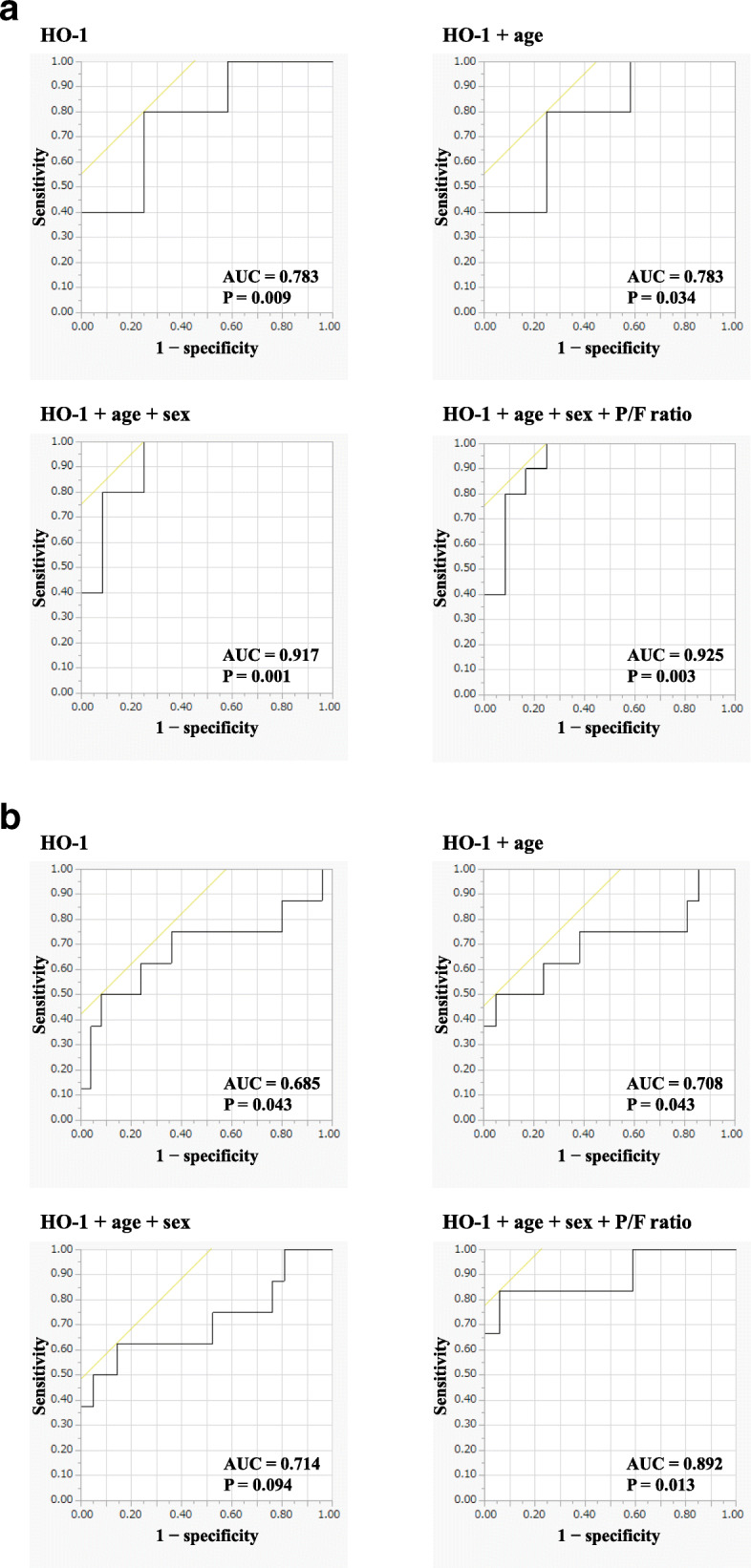
Composite parameters for predicting 3-month mortality in patients with ARDS and AE-ILDs. In both patients with ARDS and AE-ILDs, we evaluated the predictability for the 3-month mortality. In patients with ARDS, composite parameters including serum HO-1, P/F ratio, age and sex for prediction of 3-month mortality showed a higher AUC (0.925) than AUCs of a single predictor (only HO-1; 0.783) or combination of two (HO-1 and age; 0.783) or three predictors (HO-1, age, and sex; 0.917) (**a**). Also, in patients with AE-ILDs, composite parameters including serum HO-1, P/F ratio, age and sex for prediction of 3-month mortality showed a higher AUC (0.892) than AUCs of a single predictor (only HO-1; 0.685) or combination of two (HO-1 and age; 0.708) or three predictors (HO-1, age, and sex; 0.714) (**b**)

In both patients with ARDS and AE-ILDs, we evaluated the predictability for the 3-month mortality. In patients with ARDS, composite parameters including serum HO-1, P/F ratio, age and sex for prediction of 3-month mortality showed a higher AUC (0.925) than AUCs of a single predictor (only HO-1; 0.783) or combination of two (HO-1 and age; 0.783) or three predictors (HO-1, age, and sex; 0.917) (Fig. 3a). Furthermore, composite parameters including serum HO-1, P/F ratio, age and sex showed a higher AUC (0.925) than AUC of the acute physiology and chronic health evaluation (APACHE) II score which was frequently used to measure disease severity in intensive care unit patients with ARDS (AUC; 0.563) [26]. In patients with AE-ILDs, composite parameters including serum HO-1, P/F ratio, age and sex for prediction of 3-month mortality showed a higher AUC (0.892) than AUCs of a single predictor (only HO-1; 0.685) or combination of two (HO-1 and age; 0.708) or three predictors (HO-1, age, and sex; 0.714) (Fig. 3b).

## Discussion

Increased oxidative/nitrosative stress might play a major role in the progression of various lung diseases including AE-ILDs and ARDS [9–14]. HO-1, a rate-limiting enzyme in heme catabolism, has antioxidative activities in patients with these diseases [27–29]. We previously investigated whether evaluating the degree of oxidative stress by measuring serum HO-1 using the sandwich ELISA method is useful for assessing disease activities and predicting prognosis in patients with ARDS and AE-ILDs [19, 20]. In the present study which was an integrated analysis of these previous studies, we investigated whether composite parameters including serum HO-1 and other clinical parameters could predict the prognosis of ARDS and AE-ILDs more accurately than serum HO-1 alone and oxidative stress measured by serum HO-1 levels could be reduced in the clinical course.

ARDS and AE-ILDs are a life-threatening event and the mortality rate is high [30, 31]. A retrospective analysis in patients with ARDS showed a significant increase in hospital mortality in patients with DAD compared to those without DAD (71.9% vs. 45.5%) [30]. Other retrospective cohort study in patients with AE of chronic fibrosing IP showed the overall survival after admission was 67% at 1 month and 40% at 3 months [31]. In both patients with ARDS and AE-ILDs, our data showed better prognosis than these cohort studies, however, the patients with ARDS tended to show worse prognosis than those with AE-ILDs as previously reported. In the present study, ARDS patients had significantly higher serum HO-1 and CRP levels at baseline compared with AE-ILD patients and serum HO-1 had positive correlation with serum LDH. These results indicate that ARDS patients had a stronger degree of systemic inflammatory response, pulmonary epithelial cell damage and endothelial cell damage with the consequent increase of vascular permeability reflecting oxidative/nitrosative stress response than AE-ILD patients [32–34]. Though similar with HO-1, SODs, catalase, and GPx had been described as the endogenous antioxidants, HO-1 characterized as stress response protein with relatively small-molecular weight (32 kDa, much smaller than KL-6 (5000 kDa)), rapid response against stimuli, and various physiological activities including the antiapoptotic, anti-inflammatory, vasodilatory, anticoagulant, antioxidant, and antiproliferative reactions caused by HO-1 metabolites [7, 15–17]. Therefore, we speculate that serum HO-1 reflects pulmonary cellular damage induced by ROS and RNS more closely and directly.

Ongoing and persistent oxidative stress leads to poor prognosis [35]. In patients with ILDs, persistently high ethane levels, a product of lipid peroxidation that has been proposed as a biomarker of oxidative stress, may correlate with poor prognosis [35]. Cancer cells with persistent Nrf2 activation often develop Nrf2 addiction and show malignant phenotypes, leading to poor prognoses [36]. In the present study, although serum HO-1 levels tended to decrease 2 weeks after the start of treatment in both ARDS and AE-ILD patients, serum HO-1 levels remained persistently high. Furthermore, in patients treated with intravenous corticosteroids, no significant decrease of serum HO-1 levels was observed. Systemic corticosteroids are able to block nuclear translocation of nuclear factor- kB, the main pathway of inflammatory cytokine synthesis, through their interaction with the glucocorticoid receptor, however, the use of corticosteroid in ARDS is not recommended routinely [37, 38]. Also, retrospective data derived from AE-IPF patients treated with corticosteroid alone did not show any reduction in mortality rate over the short term [39]. Several evidence in the animal models suggest that corticosteroid exposure can cause to increase oxidative stress [40, 41]. Although the exact mechanism by which corticosteroid increase oxidative stress is not well known, several hypothesis have been reported that glucocorticoids could bind to mitochondrial glucocorticoid receptors and activate mitochondrial function to generate ROS or ROS is generated by the activation of the protein kinase C (PKC) β / p66^shc^ signaling pathway by glucocorticoid in the cell [40, 41]. Therefore, in patients with ARDS and AE-ILDs, the use of corticosteroids may be harmful from the point of view of oxidative stress increase, and the potential antioxidant treatment such as N-acetylcysteine needs to be examined in the future [42].

Composite approaches have been developed using peripheral blood biomarkers and physiological and radiographic measurements to provide more accurate prognostic information [26, 43, 44]. For example, APACHE II score is frequently used to measure disease severity in intensive care unit patients with ARDS [26]. The composite scoring system, which is based on serum LDH, Krebs von den Lungen-6, P/F ratio, and extent of abnormal high resolution computed tomography findings, is useful for predicting 3-month mortality in AE-IPF patients [43]. We previously demonstrated that the Charlson Comorbidity Index score, sex, and serum LDH are important for predicting 3-month mortality in AE-ILD patients [44]. In the present study, we found that composite parameters including serum HO-1, P/F ratio, sex, and age which was characterised as objective biomarkers had acceptable AUC for prediction of 3-month mortality in ARDS and AE-ILD patients. Our data suggest that composite parameters including serum HO-1 and other clinical parameters could predict the prognosis of ARDS and AE-ILDs more accurately than serum HO-1 alone. To verify the utility and reproducibility of this composite parameters, large-scale, multi-institutional prospective collaborative research is essential.

There are several limitations to the present study. First, the study enrolled only a small number of patients from a few institutions. Our findings need to be confirmed in a multi-centre, prospective study. Second, the various endogenous oxidative stress markers such as not only HO-1 but also SODs, catalase, GPx, and myeloperoxidase have been reported. It is necessary to evaluate which markers are the most reliable for predicting the prognosis of ARDS and AE-ILDs. Third, clinical diagnoses among ARDS and AE-ILD patients were heterogeneous. Actually, in both patients with ARDS and AE-ILDs, our data showed better prognosis than the previously reported cohort data. In the future, it is necessary to evaluate the clinical significance of serum HO-1 in patients with DAD histologically.

## Conclusions

Oxidative stress assessed by serum HO-1 is persistently high among enrolled patients for 2 weeks after diagnosis despite treatment with corticosteroids. Also, composite parameters including serum HO-1, P/F ratio, sex, and age had acceptable AUCs for prediction of 3-month mortality in ARDS and AE-ILD patients.

## Data Availability

The datasets used and/or analysed during the current study are available from the corresponding author on reasonable request.

## Abbreviations

AE: Acute exacerbation
APACHE: Acute physiology and chronic health evaluation
ARDS: Acute respiratory distress syndrome
AUC: Area under the ROC curve
CRP: C-reactive protein
CSE: Cigarette smoke extract
CVD-IP: Collagen vascular disease-related interstitial pneumonia
DAD: Diffuse alveolar damage
ELISA: Enzyme-linked immunosorbent assay
GPx: Glutathione peroxidase
HO-1: Heme oxygenase-1
HRCT: High resolution CT
IIPs: Idiopathic interstitial pneumonias
ILD: Interstitial lung disease
IPF: Idiopathic pulmonary fibrosis
LDH: Lactate dehydrogenase
Nrf2: Nuclear factor erythroid 2-related factor 2
P/F ratio: Partial pressure of oxygen in arterial blood/fraction of inspired oxygen
PKC: Protein kinase C
RNS: Reactive nitrogen species
ROC: Receiver operating characteristic
ROS: Reactive oxygen species
SODs: Superoxide dismutases
T-bil: Total bilirubin
